# Machine learning algorithm improved automated droplet classification of ddPCR for detection of BRAF V600E in paraffin-embedded samples

**DOI:** 10.1038/s41598-021-92014-4

**Published:** 2021-06-16

**Authors:** Gabriel A. Colozza-Gama, Fabiano Callegari, Nikola Bešič, Ana Carolina de J. Paniza, Janete M. Cerutti

**Affiliations:** 1 Genetic Bases of Thyroid Tumor Laboratory, Division of Genetics, Department of Morphology and Genetics, Universidade Federal de São Paulo, São Paulo, SP Brazil; 2 Department of Pathology, Universidade Federal de São Paulo, São Paulo, SP Brazil; 3 Department of Surgical Oncology, Institute of Oncology – Onkološki inštitut Ljubljana, Ljubljana, Slovenia

**Keywords:** Thyroid cancer, Cancer genetics, Diagnostic markers, Predictive markers, Prognostic markers, Molecular medicine

## Abstract

Somatic mutations in cancer driver genes can help diagnosis, prognosis and treatment decisions. Formalin-fixed paraffin-embedded (FFPE) specimen is the main source of DNA for somatic mutation detection. To overcome constraints of DNA isolated from FFPE, we compared pyrosequencing and ddPCR analysis for absolute quantification of BRAF V600E mutation in the DNA extracted from FFPE specimens and compared the results to the qualitative detection information obtained by Sanger Sequencing. Sanger sequencing was able to detect BRAF V600E mutation only when it was present in more than 15% total alleles. Although the sensitivity of ddPCR is higher than that observed for Sanger, it was less consistent than pyrosequencing, likely due to droplet classification bias of FFPE-derived DNA. To address the droplet allocation bias in ddPCR analysis, we have compared different algorithms for automated droplet classification and next correlated these findings with those obtained from pyrosequencing. By examining the addition of non-classifiable droplets (rain) in ddPCR, it was possible to obtain better qualitative classification of droplets and better quantitative classification compared to no rain droplets, when considering pyrosequencing results. Notable, only the Machine learning k-NN algorithm was able to automatically classify the samples, surpassing manual classification based on no-template controls, which shows promise in clinical practice.

## Introduction

Sanger sequencing is still considered the gold standard for detecting mutations when small fragments of DNA are analyzed for specific single nucleotide variants (SNVs). Although Sanger can uncover new mutations and is affordable, it has specific drawbacks. In the routine cancer histology, tissue biopsies and surgical specimens are fixed in formalin-fixed paraffin-embedded (FFPE) sections for diagnostic purposes. This procedure compromises the quality of genomic DNA and, therefore, the PCR-based analyses of DNA isolated from FFPE such as Sanger Sequencing. Additionally, it does not allow quantitative evaluation of mutated alleles, as well as presents low sensitivity for detecting somatic cancer mutations present at very low (< 20%) variant allele frequency (VAF).

In this new era, many methods derived from PCR and sequencing have been developed to detect mutations and surpass the limitations of Sanger Sequencing. One such methodology is pyrosequencing, a synthesis-based sequencing method that uses small fragments of PCR to initiate the synthesis of a new strand and detect incorporated bases by fluorescence. This methodology has vastly improved detection of SNVs, especially in highly degraded material derived from FFPE. Another advantage is that, by comparing base incorporation on specific locations of the fragment, it is possible to achieve absolute quantification of both mutated and wild-type (Wt) alleles^1,2^.

One breakthrough technology for detection and quantification of nucleic acid is the droplet digital PCR (ddPCR), which measures absolute number of targets present in the samples. One advantage of ddPCR is that it is an old technology based on new chemistry and, therefore, the platform shows great potential for advancements, such as the possibility to perform higher multiplexing and the use of machine learning methods to implement a more accurate automatic classification of droplets.

This approach is based on the partition of template DNA copies into 10,000–20,000 droplets, and the PCR reaction is carried out within each droplet. Therefore, the sample is portioned enabling the measurement of thousands of independent amplification events within a single sample, which implicates higher sensitivity. Each target is labeled with a specific fluorophore. At the end-point reactions, the droplets are then scored for the presence (positive) or absence (negative) fluorescence signals and the ratio of positive or negative droplets are then analyzed using Poisson distribution. The limiting dilution strategy and the Poisson distribution allows to determine the absolute count of target DNA copies per sample^3,4^.

However, one limitation of ddPCR is that the PCR reaction that occurs within each droplet still depends on the quality of the DNA. Although ddPCR requires smaller amount of input DNA and a smaller amplicon size, the DNA isolated from FFPE sections is highly degraded and contain PCR inhibitors. Therefore, not all partitions with the target DNA will amplify at the same rate and some droplets that contain the target sequences may exhibit reduced fluorescence and will be considered negative. When duplex ddPCR is used, i.e., two fluorescence probes are used, droplets can be classified as positive for both fluorescence (PP), positive for just one fluorescence (NP or PN), containing no fluorescence (NN) and those with fluorescence ranging between those of unequivocal positive and negative droplets (rain effect). The origin of rain droplets could be a result of damaged positive droplets with diminished fluorescence, damaged negative droplets with increased background fluorescence, partial PCR inhibition in same droplets and delayed PCR start. The drawback is that presence of rain can interfere in the analysis and the correct measurement of droplets. This is particularly true for quantification of somatic mutations in cancer. Regarding the Biorad’s ddPCR software, it is unfeasible to classify FFPE samples droplets automatically. The main indication in this case is to classify manually the droplets based on a negative control without DNA.

Many algorithms have been developed to help automatically classify these droplets as positive, negative, or unclassifiable “rain” droplet. Perhaps one of the very first proposed algorithms is “definetherain”^5^, which can be used for uniplex analysis. Several other algorithms, which seek to automatically classify these droplets, but for duplex or even higher order of multiplex reactions, are now available. To verify which methodology would be the best suited for absolute quantification of BRAF Wt and mutant V600E alleles in the DNA isolated from FFPE specimens, in this manuscript we initially employed pyrosequencing and ddPCR analysis and compared the results to the qualitative detection information obtained by Sanger Sequencing. Next, to overcome the droplet allocation bias in ddPCR analysis, we compared different algorithms for automated droplet classification and correlated these findings with those obtained from pyrosequencing. We selected the k-NN machine-learning algorithm as the best to automatically classify the droplets.

## Results

### Pyrosequencing is a reliable method to detect BRAF V600E point mutation in DNA isolated from FFPE sections

As controls, we used DNA isolated from FFPE sections of a papillary thyroid carcinoma (PTC) and a follicular thyroid adenoma (FTA) known as positive and negative for BRAF V600E, respectively. To classify a sample as positive or negative, allele percentage threshold of BRAF V600E was established in positive and negative controls. Based on results obtained in negative control, a cut-off of 9.82% was established (Fig. S1). Any value above this threshold was then considered positive for the BRAF V600E mutation. Next, all papillary thyroid microcarcinoma (microPTC) and lymph node metastases samples were screened for BRAF V600E mutation by pyrosequencing. Using the threshold of 9.82%, 103/115 (89%) samples were positive for BRAF V600E. To measure the precision of the results we randomly selected 23 samples and repeated the pyrosequencing analysis. The result was consistent for all re-sequenced samples, demonstrating accuracy and reproducibility of pyrosequencing.

### Limits of detection of BRAF V600E allele by Sanger and pyrosequencing

**Figure 1:**
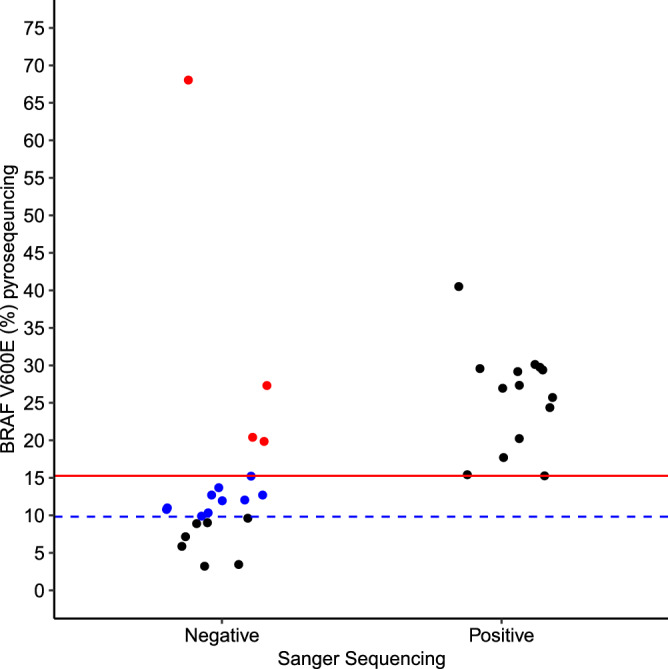
Limits of detection of BRAF V600E allele for pyrosequencing and Sanger (*n* = 35). The red line represents the observed limits of detection of BRAF mutated allele by Sanger (> 15%). The doted blue line represents the threshold (> 9.82%) used to define a sample as positive for BRAF V600E mutation by pyrosequencing. Pyrosequencing detected BRAF V600E mutation in 10 samples (blue dots) that were negative by Sanger. Four samples were positive by pyrosequencing (red dots) but were negative by Sanger, even if the percentage of mutant allele was greater than 15%.

Consistent with the findings in the literature, all samples positive for BRAF V600E by Sanger presented at least 15% of mutant allele (red line), in the background of Wt allele (Fig. 1). The limit of detection of BRAF mutant allele by pyrosequencing (doted blue line) was superior (> 9.82%). Hence, all samples considered positive for BRAF V600E mutation by Sanger were also positive by pyrosequencing.

Discordant results were observed for 14 samples, which were negative for BRAF V600E mutation by Sanger but were classified as positive by pyrosequencing (Fig. 1). Remarkable, in 4 samples that were positive by pyrosequencing but were negative by Sanger (red dots), the percentage of mutant allele was greater than 15%, which would be expected to be detectable by Sanger as it is above the limit of detection of that technique. These findings suggested that Sanger was suboptimal not only when low frequencies of mutant alleles were observed. As the DNA was isolated from FFPE and some times it can be highly degraded, Sanger sequencing failure is probably related to the large size of PCR products. The remaining 10 samples that were negative by Sanger (blue dots), in fact were under the limited detection of Sanger but within the limit of detection by pyrosequencing. These findings confirmed that pyrosequencing has a higher detection rate than Sanger.

### Adding rain to droplets classification of ddPCR increase accuracy of BRAF V600E detection

**Figure 2:**
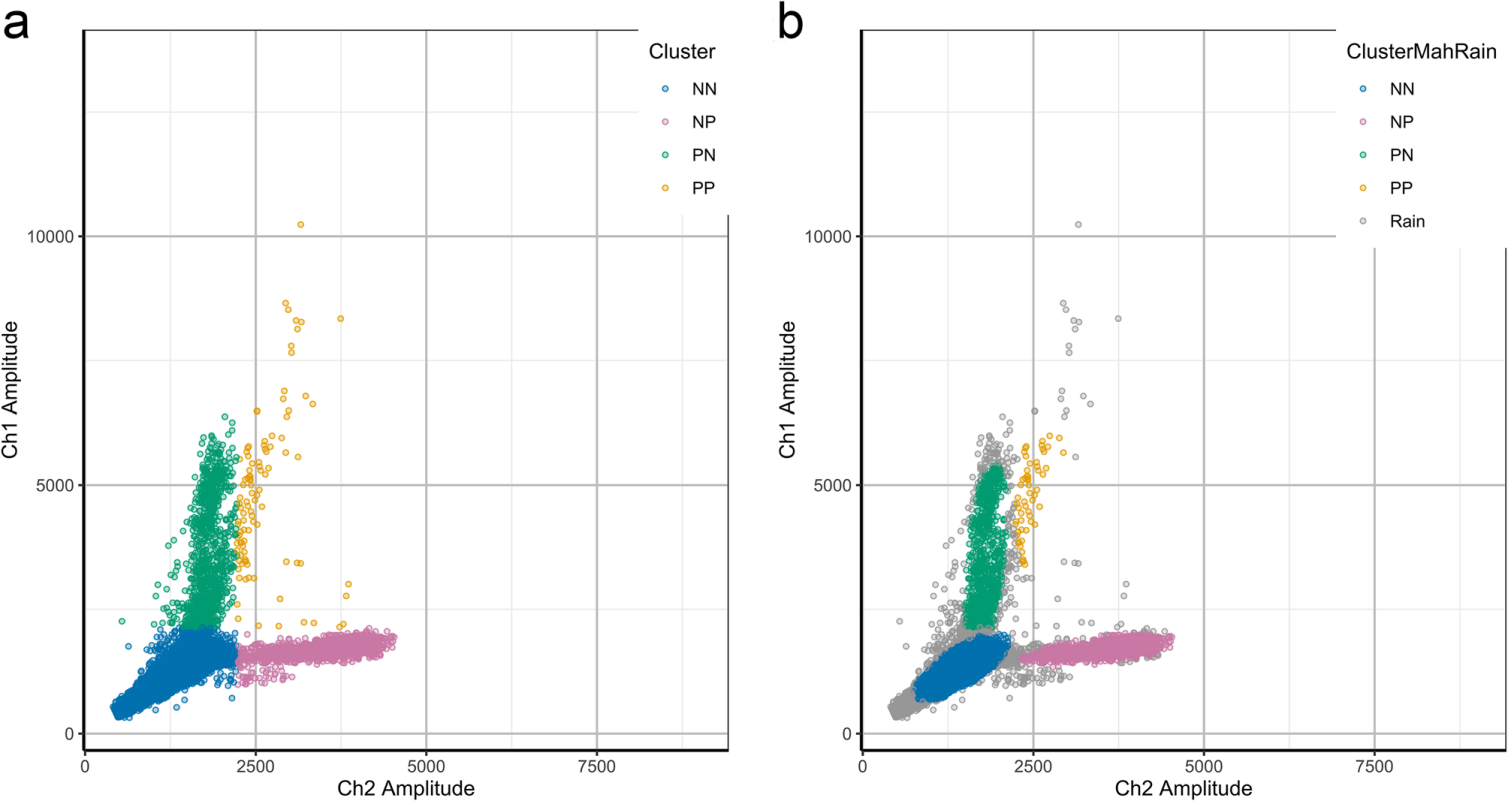
Manual classification of ddPCR overlooking rain droplets or excluding rain droplets. Representative results of the analysis of 21 samples and the negative and the positive controls. All droplets are combined into a single plot. The channel 1 axis represents mutation-specific Taqman probe (VIC fluorescence) and channel 2 axis represents a wild-type Taqman probe (FAM fluorescence). (**a**) Plot with classification of the droplets without exclusion of “rain droplets”. It is possible to observe that all droplets are classified in four groups. (**b**) Plot with classification of the droplets after exclusion of “rain droplets”. It is possible to observe that extreme droplets were removed, including broken droplets, which have very low fluorescence in both axes. Droplets that were hard to classify, as they are very close to one or more clusters, were also removed. NN = No DNA; NP = positive for wild type BRAF; PN = positive for BRAF V600E; PP = positive for both wild type and mutated BRAF; Rain = droplets hard to classify, excluded from the analysis.

To compare the ddPCR sequencing results to both Sanger and pyrosequencing, we first classified the droplets. Although there are many ways of classifying droplets, it mostly consists in detecting clusters of droplets that are positive for Wt, positive for BRAF V600E mutation, positive for both alleles or negative for the target DNA. There are regions between clusters where the classification of the droplets is ambiguous, as they are not empty but the ddPCR reader did not detect enough fluorescence emitted from them. Additionally, there are droplets under the limit of no DNA detection that might be broken/damaged droplets and droplets which have unusually high fluorescence. All these droplets can be termed “rain”, which are particularly prevalent in experiments conducted on DNA isolated from FFPE^5^ and should be removed from the analysis. For this reason, the parameters should be adjusted in order to remove those from the droplet counts in each of the clusters (Fig. 2). To investigate if adding rain to droplets classification of ddPCR improves the data, some assumptions are needed: droplets with high values can contain more than two molecules of DNA and should be removed; droplets with values that are too low might be broken droplets, which also should be removed; droplets which are too close to other clusters cannot be classified with confidence and should be removed. As shown in Fig. 2, the exclusion of droplets that are hard to classify in panel b (rain droplets), which influence on the qualitative results, increased the value of classification based in all the previous assumptions.

**Figure 3:**
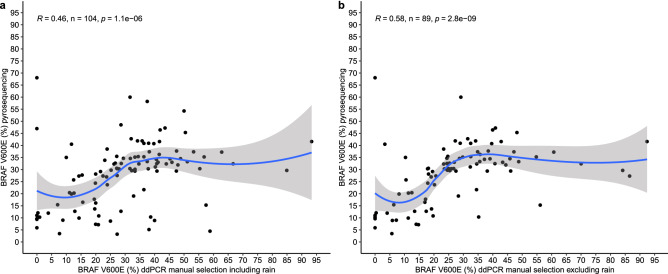
Comparison of the percentage of BRAF V600E mutated allele by ddPCR following manual classification of rain droplets or pyrosequencing. (**a**) Comparison of pyrosequencing with ddPCR following inclusion of rain droplets. This graph shows that some samples have different percentages of BRAF V600E mutated allele if the rain droplets (non-classifiable droplets) were included. (**b**) Comparison of pyrosequencing with ddPCR excluding rain droplets. In this analysis, the confidence interval is squeezed and less extreme values are present, showing that the exclusion of rain droplets soothes lightly the distribution of values. The exclusion of the rain droplets increases the correlation between ddPCR and pyrosequencing. The blue line shows the result of LOESS regression and the gray area shows the 95% confidence interval of the regression. R, N and P are calculated for Spearman correlation test.

Additionally, the percentage of BRAF V600E alleles obtained via ddPCR and manual classification of droplets were compared with those values obtained from pyrosequencing analysis (Fig. 3). Based on its sensitivity, accuracy and reproducibility, pyrosequencing was chosen as the gold standard method to detect BRAF V600E mutation.

The results show that excluding rain droplets increases the correlation between ddPCR and pyrosequencing, as observed by increased spearman correlation values. These findings suggest that excluding rain droplets increases the accuracy of ddPCR when absolute quantification is taken into account, as it minimizes variation of samples.

### The k-NN algorithm is better than manual classification of droplets

Of note, the automatic analysis of the droplets failed with ddPCRclust package^6^ and ddPCR package^7^ for all our samples (results not shown). On the other hand, the k-Nearest Neighbors (k-NN) algorithm in twoddPCR package, which is based on lazy machine learning, was the only one that successfully classified the droplets.

**Figure 4:**
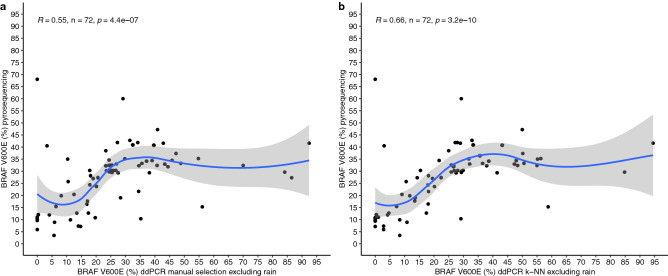
Comparison of the percentage of BRAF V600E mutated allele by pyrosequencing and ddPCR following manual droplet selection or automatic classification using k-NN algorithm. (**a**) Comparison of ddPCR with manual classification excluding rain droplets and pyrosequencing. (**b**) Comparison of ddPCR with automatic classification using the k-NN algorithm excluding rain droplets and pyrosequencing. All 18 samples used in the training of k-NN algorithm were removed from the analyses of both panels. The spearman correlation test in panel (**b**) (R = 0.66, *p* < 0.05) shows that k-NN algorithm values are closer to the values of pyrosequencing, even though the droplets were classified automatically based on the training dataset. When compared to manual selection on panel a (R = 0.55, *p* < 0.05), the results of automatic classification are satisfactory.

Therefore, we next compared the percentage of BRAF V600E mutation measured by pyrosequencing and ddPCR with manual selection of droplets or automatic classification of droplets using the k-NN algorithm. As is recommend with most machine-learning methodologies, we removed the training dataset consisting of 18 samples from all comparative analysis, where the results are shown in Fig. 4.

Remarkable, the outcome of the ddPCR followed by supervised machine-learning algorithm showed a better correlation with those results from pyrosequencing than the results of the ddPCR with manual gating, even when rain analysis was made. While manual selection obtained a statistically significant correlation (R = 0.55, *p* = 4.4^−07^), this score was improved with k-NN (R = 0.66, *p* = 3.2^−10^). Using pyrosequencing as our gold standard, this correlation shows that better results can be obtained with k-NN. Moreover, the automatic detection must improve user selection bias drastically, as there is no need for manual selection of clusters of droplets after the training dataset for k-NN is classified and used.

### Evaluation of ddPCR with k-NN algorithm for qualitative BRAF V600E detection

**Figure 5:**
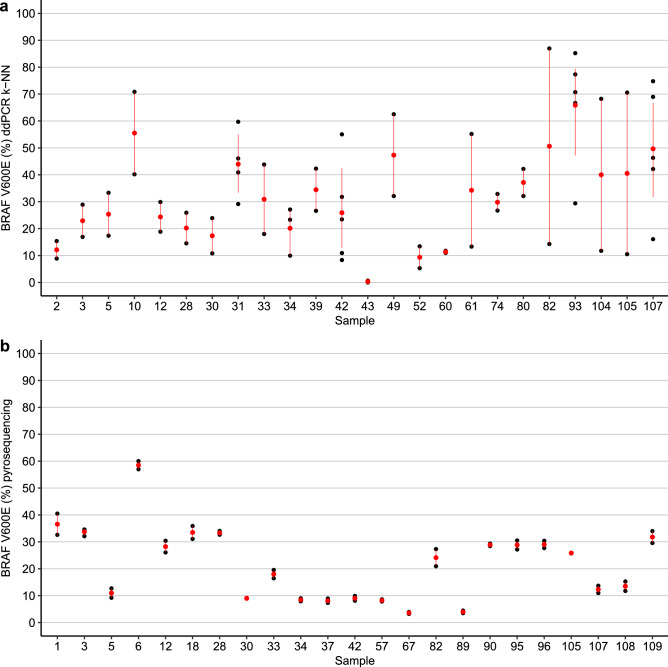
Comparison of digital PCR and pyrosequencing repetitions. Each black dot consists of one repetition, the red dot is the mean value of repetitions and the red line represents the standard deviation. (**a**) Digital PCR percentage of BRAF V600E when using k-NN classification method with rain. The repetitions, in general, have a high variability. The mean of the standard deviation of each repetition is 10.62. Adopting the threshold of 9.82% mutated alleles, some samples are considered positive or negative depending on the repetition. This is true for samples 52, 2, 42 for example. (**b**) Pyrosequencing BRAF V600E percentage. Overall, any sample repeated in pyrosequencing was very close to one another in terms of BRAF V600E VAF. The mean of the standard deviation of each repetition is 1.32, which is very low. Additionally, only one sample, with the highest value of the repetition being 9.9%, changed classification of positive or negative based on the threshold of 9.82%.

When comparing ddPCR with k-NN classification algorithm, BRAF V600E was detected in two samples that were negative for pyrosequencing and Sanger, even after multiple tests, suggesting that ddPCR can be more sensitive than conventional Sanger and pyrosequencing (Fig. 5). All samples detected by Sanger were also detected by ddPCR with k-NN and pyrosequencing.

Unexpectedly, 10 samples considered positive by pyrosequencing were negative by ddPCR (Table 1). Although the ddPCR has unique features that make its superior to conventional PCR and Sanger Sequencing and it is robust to many of the factors that can negatively influence conventional PCR, one reasonable explanation for the negative samples is that FFPE tissue-derived DNA is highly degraded and can contain PCR inhibitors.

**Table 1:** Discordant BRAF V600E status relative to the pyrosequencing data.

	ddPCR k-NN algorithm	Sanger sequencing
Discordant	12	14
Positive	2	0
Negative	10	14

It has been reported that ddPCR is less prone to “allelic drop out” phenomenon commonly observed in conventional PCR and Sanger Sequencing because it requires lower input of DNA and smaller amplicon sizes. Although we do not have a definitive explanation for this, the PCR failure in these samples is probably related to the longer PCR amplicon size for ddPCR (130pb) than the PCR amplicon length for pyrosequencing (119pb). Additionally, 8 out of 10 negative samples by ddPCR showed the percentage of mutated alleles very close to the threshold of 9.82% determined for pyrosequencing. As the ddPCR product was larger than the PCR product used for pyrosequencing and the percentage of BRAF mutated allele was low we presumed that the target DNA molecule might be degraded, which could explain our results.

The remaining two samples showed 68% and 40% percentage of mutated allele by pyrosequencing. Although the proportion of mutated allele was high, these two samples had lower initial DNA concentration and were not further diluted, as the volume of DNA template for ddPCR reaction cannot exceed 8.5 µL. Because for these two samples more starting material was needed to achieve the required total DNA and the PCR reaction was performed in a smaller final volume than the final volume of the PCR reaction used for the pyrosequencing, we hinted to the hypothesis that contaminants originating from the FFPE process were present in these samples, low yield or low quality DNA could have affected the sensitivity of the methodology as isolated fragment size and residual crosslinks are key determinants of downstream assays.

All together, these could explain, at least in part, why we had larger variability to detect the mutation by ddPCR with k-NN algorithm (Fig. 5). Therefore, we emphasise that very short amplicon sizes are recommended for FFPE-derived DNA.

### Comparison of digital PCR and pyrosequencing for quantitative BRAF V600E detection

As both ddPCR and pyrosequencing have high sensitivity to detect single nucleotide variants, one of our goals was to evaluate if the techniques had any difference in terms of specificity. A total of 23 samples were repeated at least twice for pyrosequencing and 24 samples were repeated at least twice for ddPCR.

For DNA isolated from FFPE tissue, the repetition of assays showed that pyrosequencing had less variability than ddPCR. The maximum difference between repetitions of the same sample for pyrosequencing was 7.9% and the mean difference was 2.7%. However, for ddPCR and the k-NN algorithm, the difference was up to 72.69% with a mean difference of 26.59% (Fig. 5).

Another important point is that the mean standard deviation for repetitions was 12.72 for ddPCR with manual classification and exclusion of the droplets labeled as rain, 10.72 for ddPCR with k-NN algorithm and exclusion of rain droplets, and only 1.32 for pyrosequencing. This result shows that k-NN reduces standard deviation in replicates, while pyrosequencing have a very low standard deviation from replicates in general. Thus, selecting pyrosequencing as our gold standard seemed the most appropriate, even though both techniques showed similar sensitivity.

**Figure 6:**
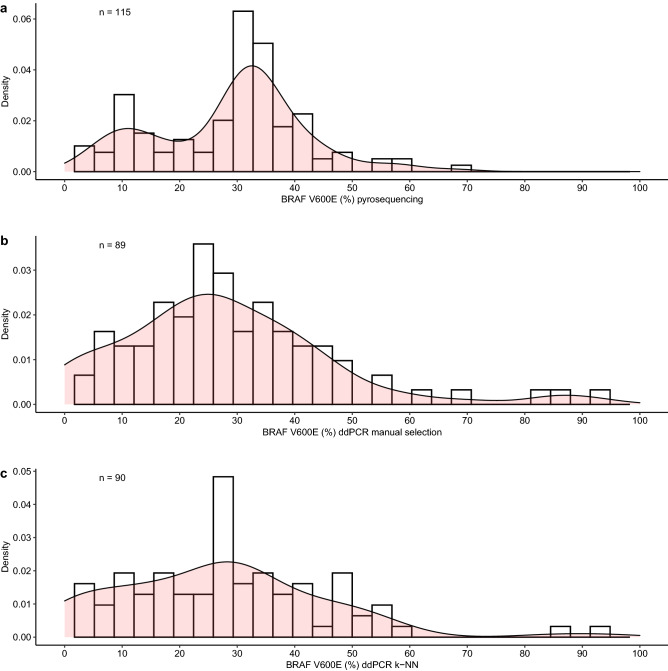
Distribution of percentage of BRAF V600E by using different techniques. The density plot represents the distribution of BRAF V600E percentage across all samples. It uses a kernel density estimate to show a fitted density curve. Not only, but it also shows the histogram of this distribution. (**a**) Percentage obtained through pyrosequencing. There is the formation of two populations, one with lower percentage (< 20%) with the center close to 10% and one with higher percentage (> 20%) with the center close to 34%. (**b**) Percentage obtained through manual selection excluding rain droplets in ddPCR. (**c**) Percentage obtained through k-NN selection excluding rain droplets in ddPCR. It is possible to check that for both ddPCR graphs, only one curve of distribution seems to be clearly visible. Not only, but the density curve is smoothed in k-NN values, with less extreme values close to 100% BRAF V600E.

To further explore how the different ddPCR droplet analysis compares to pyrosequencing, a full description on the distribution of BRAF V600E percentage was obtained and it is shown in Fig. 6.

If we assume that there are, in general, one population of tumors without BRAF V600E and one population with BRAF V600E, we should expect at least two clearly separated populations when analysing the VAF of samples given the presence of the mutation in homozygosis or heterozygosis.

In fact, it is possible to observe that for pyrosequencing analysis, two populations are undoubtedly observed. Considering that the threshold for BRAF V600E positivity is 9.82%, the population with low abundance of mutated allele (< 10%) was considered negative and another population with a higher percentage of mutated alleles (> 10%) was considered positive.

This separation of populations is smoothed out in both ddPCR with manual selection or k-NN algorithm as shown in Fig. 6 panel b and c. Our hypothesis is that because of the high variability of the technique, the information about biological populations of tumors with different VAFs is smoothed and information is lost.

## Discussion

To our knowledge, this is the first study that compared Sanger, Pyrosequencing and ddPCR with a real-life dataset consisting of tumor samples derived from FFPE with variable DNA quality. When comparing these techniques, most studies used high quality DNA samples or even FFPE samples that were recently processed according to specific protocols and usually using a serial dilution of a high-quality sample to quantitative comparison^1,2,5–10^. Although there are studies in the literature that compared pyrosequencing with Sanger or ddPCR, as far as we know, there is no study that evaluated absolute quantification of somatic mutations in FFPE tissue-derived DNA and in general studies select the classification that found the most positive qualitative results for SNP detection^1,9^.

We here aimed to identify strategies that would give us the most reliable results to detect BRAF V600E mutated allele in DNA isolated from FFPE specimens.

In addition to detect the presence/absence of mutated allele, the VAF detections likely have an important effect on biological behavior of the tumor. In the literature, many works have shown that tumors with higher VAF of some drivers such as P53 have worse prognosis and disease-free survival^11–13^. In PTC with tumor size > 1 cm, it has been shown that when more than 30% of BRAF V600E is present there is an association with disease aggressiveness and poorer outcome^14^. Moreover, some diagnostic techniques such as fine-needle aspiration (FNA) and conventional biopsies may contain a high number of infiltrate cells and, therefore, sensitive detection of mutations are important for both clinical practice use and research^15^.

One example is that if BRAF V600E is found in cells obtained from FNA of a thyroid nodule, even within low mutated allele frequency, the nodule is considered malignant and surgery is indicated based on this finding^16^. Another important point to the use of quantitative allele detection is that some mutations, even when present at very low abundance, triggers important and deleterious effects, such as mutations related to tumor progression and drug resistance^17,18^.

This fact encourages the use of quantitative techniques for the detection of variant allele frequency in genes related to cancer.

The need for quantitative sequencing technologies for genetics disease such as cancer is one reason for the development of new technologies. One example is digital PCR, which has been proposed some years after the conception of conventional PCR. Nevertheless, the procedure of creating thousands of separated reactions was unfeasible with the technology. Recently, the correct chemistry was improved to allow the generation of droplets from the same PCR reaction, by using microfluidics. Droplet digital PCR can be very helpful, with high precision and sensitivity, but the challenges to include this technology in clinical environment are not elementary. As an example, the hands-on is vastly more complicated than qPCR, Sanger Sequencing and pyrosequencing. Additionally, the initial DNA input varies according to the platform used which should be considered for rare samples such as microPTC. Lastly, there is no obvious method for the automatic classification of positive and negative samples for degraded genetic material such as that obtained from FFPE.

When comparing the three different approaches to detect a somatic driver mutation, we observed that, when using DNA isolated from FFPE specimens, Sanger Sequencing showed lower accuracy and sensitivity than ddPCR and pyrosequencing to detect BRAF V600E mutation. In fact, these comparisons have been independently performed and suggested that Sanger Sequencing is not as sensitive as ddPCR and pyrosequencing^1,2,9^. Therefore, our data corroborate with this hypothesis and confirmed that Sanger require at least > 15% of abundance of mutated allele to detect a mutation as seen in Fig. 1.

As the percentage of mutated alleles in few samples varied between ddPCR analyses, our hypothesis is that ddPCR was sensitive to amplicon size. As FFPE-derived DNA is highly degraded, its sensitivity is extremely dependent of primer and probe designs that affect the size of PCR product. Additionally, we cannot exclude that inhibiting substances were co-eluted during DNA isolation and were present at higher concentrations in few original samples and could contribute to PCR failure. Therefore, smaller amplicon size and lower input of FFPE tissue-derived DNA are critical. One recommendation for FFPE tissue in ddPCR is that rare samples with very low DNA concentration could be evaporated by using vacuum concentrator so no DNA is lost, and FFPE contaminants could be eluted when water is added to the ddPCR reaction mix to complete 20ul final volume.

Remarkable, pyrosequencing and ddPCR were able to classify a sample as positive when only 10% of mutated alleles are present. Importantly, there is a lot of room for the improvement of ddPCR, which has begun to be used in the clinical setting in recent years, while the pyrosequencing is virtually closed for new developments.

One advantage of ddPCR over pyrosequencing is that ddPCR is an old technology based on new chemistry and, therefore, the platform shows great potential for advancements, such as the possibility to perform higher multiplexing and the use of machine learning methods to implement a more accurate automatic classification of droplets.

One example that needs improvement is to overcome the droplet allocation bias in ddPCR analysis. However, most algorithms used to automatically classify droplets were tested using serial dilution of one sample and did not compare to other techniques that are also sensitive such as pyrosequencing^6–8^. To fill this gap, we sought to compare classification algorithms with the most reliable method that we found, which was pyrosequencing.

In our study, we could compare the results of clinical samples with another already proven technology. First, machine learning was able to automatically classify the experiments made with FFPE-derived DNA. Additionally, we found that the automatic classification of droplets could increase the sensitivity and precision of this technique when comparing with pyrosequencing (Fig. 4), but also reduce selection bias. Nevertheless, one weakness of our study is that we did not include a serial dilution of FFPE-derived DNA with high percentage of BRAF V600E mutated allele and another with low percentage of BRAF V600E mutated allele. However, there are studies showing that both techniques have a good accuracy with synthetic serial dilutions^1,2,8,9^.

Additionally, as most laboratories would not have time and resources to process samples using different techniques, providing an automated way to check for the presence of mutations is essential for clinical uses of ddPCR. With the creation of an initial training dataset of controls in ddPCR, for example, all other reactions could be run without the need to reclassify samples if k-NN classification was used.

Another important point is that simple supervised machine learning, the k-NN algorithm, can be used with very simple software in R or even online with a simplified and friendly website with the help of Shiny-based GUI^8,19^. By using the package twoddPCR, seamless integration of Biorad’s Quantstudio software can be done and automated. In principle, this algorithm is so reasonably simple that it could be easily integrated in the original ddPCR Quantstudio software.

In sum, our tests indicate machine learning is able to disentangle several pitfalls in the ddPCR droplet classification and allow broad generalization of droplet automatic classification for clinical settings. Additional machine learning algorithms should be tested with real biological samples, but even k-NN, which is very simple, showed promising results to improve ddPCR results in comparison with pyrosequencing.

## Methods

### Patients

Formalin-fixed paraffin-embedded (FFPE) sections were selected from patients who underwent thyroid surgery at Hospital São Paulo, Universidade Federal de São Paulo and diagnosed with microPTC, defined by the World Health Organization as a PTC of 10 mm or less in the largest dimension. The series consisted of 73 microPTC cases. All hematoxylin and eosin-stained slides of each sample were reviewed by a pathologist (FC), to confirm the diagnosis. For multifocal microPTC, distinct tumors foci were selected. An optimal block was selected from each case and at least one area of microPTC was selected for macrodissection. For metastatic microPTC, paraffin blocks from 20 available paired lymph node metastasis of 13 patients were macrodissected. Hence a total of 115 thyroid specimens were analysed. As controls, we additionally included FFPE from thyroid samples that had previously been confirmed as positive (PTC) and negative (FTA) for BRAF V600E mutation by Sanger Sequencing^20–22^. The study was conducted under the approval of the Research Ethical Committee of the Universidade Federal de São Paulo, São Paulo, Brazil.

### Macrodissection and DNA isolation

One H&E stained slide along with 5–8 unstained sections (five microns in thickness) were mounted on slides. Areas of interest were circled on the H&E slide by a pathologist and corresponding areas from the unstained slides were manually macrodissected using a razor blade, to remove contaminating normal cells. The paraffin fragments were placed in a 1.5 ml microcentrifuge tube, deparaffinized with xylene, vortexed, and centrifuged at 14,000 rpm × 5 min. The tissue pellet was washed twice with 100% ethanol. The DNA was extracted from the macrodissected sections using the kit GeneRead DNA FFPE tissue kit (Cat # 180134, Qiagen GmbH, Hilden, Germany) according to the manufacturer's instructions. The isolated DNA was quantified using a NanoDrop 2000c spectrophotometer (NanoDrop Technologies, Wilmington, DE, USA).

### Pyrosequencing to detect BRAF V600E mutation

Pyrosequencing was performed in all thyroid samples (*n* = 115) and controls (*n* = 2). For quantitative measurement of mutation in codon 600 in exon 15 of BRAF, a 101-bp region spanning the hotspot mutation was amplified by polymerase chain reaction (PCR). The PCR reaction was performed using a custom pyrosequencing kit from Qiagen (Cat # 979009, 979006, 970802, 979008, 978703 and 978776, Qiagen) according to the manufacturer's instructions with 10–20 ng of DNA in a final volume of 25 µL. Primer sequences were: forward: 5′-TGAAGACCTCACAGTAAAAATAGG-3′; reverse: 5′-ACAAAATGGATCCAGACAACTG-3′. The reverse primer was 5′-biotinylated to enable single-strand DNA template isolation, using streptavidin-coated sepharose beads, which is the template for the pyrosequencing reaction. The amplicons are immobilized on streptavidin sepharose high performance (Cat # 17-5113-01, GE Healthcare, Little Chalfont, United Kingdom). The single-strand DNA was sequenced using the following primer: 5′-GTGATTTTGGTCTAGCTAC-3′. The samples were then analyzed on the PyroMark Q24 and PyroMark Q24 Software (Qiagen GmbH).

Twenty-three randomly selected thyroid samples were re-analyzed, to assess reproducibility. As pyrosequencing has been largely used and considered reliable, more accurate and sensitive^1,2^ method for detection and quantification of both mutated and wild-type alleles than conventional sequencing methods, it was selected as gold standard for all analyses. To classify a sample as positive or negative, we defined the allele frequency threshold using the DNA isolated from positive and negative controls (Figure S1). Positive and negative controls were selected for their consistent results by Sanger after numerous sequencing analyses and were included in each run.

### Sanger sequencing to detect BRAF V600E mutation

All samples with less than 20% abundance of the BRAF V600E mutated allele by pyrosequencing were screened by sequenced by Sanger Sequencing as previously described^20–22^. Additionally, we randomly selected samples with > 20% of abundance of mutated alleles for Sanger Sequencing. Briefly, PCR reactions were performed using 10 ng of DNA and the amplification conditions were optimized as follows: denaturation for 10 min at 95 °C and 45 cycles of amplification. The PCR products were resolved by electrophoresis, purified, submitted to sequencing using a BigDye Terminator v3.1 cycle sequencing kit and analyzed using an ABI PRISM 310 Genetic Analyzer (Applied Biosystems, Foster City, CA, USA). The samples were sequenced at least twice and in both directions.

### Droplet digital PCR to detect BRAF V600E mutation

For Droplet Digital PCR analysis the Bio-Rad QX100 System was used in combination with the dual-probe TaqMan assay for detection of BRAF V600E mutation (Cat # Hs000000004_rm, Thermo Fisher Scientific, Waltham, MA, USA). The assay includes a mutation-specific Taqman probe (VIC fluorescence) and a wild type Taqman probe (FAM fluorescence) direct to the same region, and PCR primer pairs to amplify the sequence of interest. The PCR reaction was performed using 40–60 ng of DNA, 1× ddPCR Super Mix, 1× TaqMan assay and restriction enzyme EcoRI-HF at the concentration of 0.375U per sample (#Cat R3101, NEB, Ipswich, Massachusetts, EUA) to a final reaction volume of 20 µL. The PCR mixture was loaded into plates and the droplets were generated with droplet generation oil in the droplet generator of the QX100 system (Bio-Rad, Hercules, CA, USA). The droplets were transferred to a new plate, sealed and cycled, using the following conditions: 95 °C for 10 min, and 45 cycles of 94 °C for 30 s, and 55 °C for one minute. After PCR, the plates were placed in the droplet reader from the Bio-Rad QX100 System (Bio-Rad) and the droplets were analyzed according to the manufacture’s recommendation. Briefly, the amplified DNA in each droplet was measured for target DNA via fluorescence signaling, such that the number of positive and negative droplets can be counted. A positive and a negative control were included in each run. We additionally included a no-template control (NTC) well with no DNA. All plates were run in “rare event detection” program as suggested by BioRad. The ddPCR analysis was performed in all samples (*n* = 115). Because of the filtering changes according to droplet classification, including NTC droplet classification, analyses have different number of accepted samples. The maximum number of accepted samples was 104 samples, on manual selection without rain. The size of PCR products (approximately 130 bp) was verified using high-resolution agarose on a gel electrophoresis. The full-length gel is presented in Figure S2. The gel image was obtained with Gel Doc™ EZ System and the software ImageLab (Bio-Rad, Hercules, CA, USA) with high-intensity bands exposure.

### Droplet Digital PCR manual data analysis

The absolute quantification of mutant and wild type alleles by ddPCR was estimated by modelling as a Poisson distribution using QuantaSoft v1.7.4.0917 software (Bio-Rad). As recommended by Bio-Rad, for FFPE samples, automatic detection of probes fluorescence should not be used. Therefore, the manual selection of droplets classification was performed using the system grid classification within the limits of detection of NTC well. The discrimination between droplets was based on the signals measured in two channels, each one corresponding to the targets (BRAF V600E and BRAF wild type): Positive in both BRAF wild type and BRAF V600E (PP); positive for BRAF V600E (PN); positive for BRAF wild type (NP); and negative in both channels (NN). Then the percentage of mutated allele in each sample was evaluated.

A simple cut-off criteria for the exclusion of a reaction from subsequent analysis in all classification methods consisted of three steps: (1) reactions that had less than 10,000 acceptable droplets; (2) reactions in which the mean of positive droplets (PP, PN, and NP) was lower than the mean of positive droplets in the no-template control; (3) if more than one reaction was done, the percentage which had been the closest to the pyrosequencing percentage value was selected to be evaluated.

### Droplet Digital PCR automatic data analysis

For automatic detection of ddPCR, three packages that can analyze and visualize ddPCR data in R and are free to use and available online were used ddPCRclust package^6^, ddPCR package^7^, and twoddPCR package^8^.

When using the two ddPCR package, rain classification was added, where “rain” droplets that are hard to classify are excluded from the analysis by a defined distance. Those droplets are hard to classify because they are very close to two or more clusters. The rain distance parameter was adjusted manually according to classification results and then the same distances were used on all analyses. Also, for k-NN algorithm, the distance three (k = 3) was selected.

### Dataset for k-nearest neighbors (k-NN) algorithm classification

Because k-NN is a supervised machine-learning algorithm, a training set that had been classified manually was needed. To reduce bias, three samples were randomly chosen in each experiment, for a total of 18 samples in six different plates. These three samples should not have been excluded by the cut-off described above and should have minimal noise. Selected samples were easy to classify, but also it seemed to represent the expectations on what a good result with many droplets in both alleles is. The manual classification results that were used as the training dataset are shown in Figure S3.

### Statistics and comparison of techniques

All graphs, functions and statistics were calculated by using R version 3.6.4 and R Studio version 1.1.383 if not otherwise cited. The histogram and density graphs were obtained with the package ggplot2 and the included smoothed density estimates function was used to calculate kernel density estimate.

For comparison between percentage of BRAF V600E obtained through the classification methods of ddPCR droplets and pyrosequencing, LOESS regression with the formula $x\sim y$ and 95% confidence intervals was used as a visualization tool and spearman correlation with R values was used for statistical evaluation. A *p* value less than 0.05 (< 0.05) was considered statistically significant.

For the qualitative analysis of pyrosequencing, ddPCR and Sanger, we first classified the samples as positive or negative for BRAF V600E. To do that, the presence of BRAF V600E according to pyrosequencing and ddPCR were based in a specific cut-off. This cut-off was calculated as the upper bound of the mean 99.98% confidence interval (based on T-distribution values) of the percentage of BRAF V600E mutation in the negative controls obtained with pyrosequencing, for a very strict cut-off, as seen in Figure S1.

## Supplementary Information

Supplementary Figures.

## Data Availability

All data generated or analyzed during this study are included in this published article and its Supplementary Information files.
