# Author Correction: Machine learning algorithm improved automated droplet classification of ddPCR for detection of BRAF V600E in paraffin-embedded samples

**DOI:** 10.1038/s41598-021-94985-w

**Published:** 2021-08-02

**Authors:** Gabriel A. Colozza-Gama, Fabiano Callegari, Nikola Bešič, Ana Carolina de J. Paniza, Janete M. Cerutti

**Affiliations:** 1 Genetic Bases of Thyroid Tumor Laboratory, Division of Genetics, Department of Morphology and Genetics, Universidade Federal de São Paulo, São Paulo, SP Brazil; 2 Department of Pathology, Universidade Federal de São Paulo, São Paulo, SP Brazil; 3 Department of Surgical Oncology, Institute of Oncology – Onkološki inštitut Ljubljana, Ljubljana, Slovenia

Correction to: *Scientific Reports* 10.1038/s41598-021-92014-4, published online 16 June 2021

The original version of this Article contained an error in the spelling of the author Ana Carolina de J. Paniza which was incorrectly given as Ana C. de J. Paviza.

The original Article has been corrected.

