# Supplementary material for: Machine learning algorithm improved automated droplet classification of ddPCR for detection of BRAF V600E in paraffin-embedded samples
Source: Sci Rep. 2021 Jun 16;11:12648. doi: 10.1038/s41598-021-92014-4 (PMC8209227; doi:10.1038/s41598-021-92014-4)
Supplement: Supplementary file 1 — Supplementary Figures. [file 41598_2021_92014_MOESM1_ESM.docx]

**Supplemental figures**

**
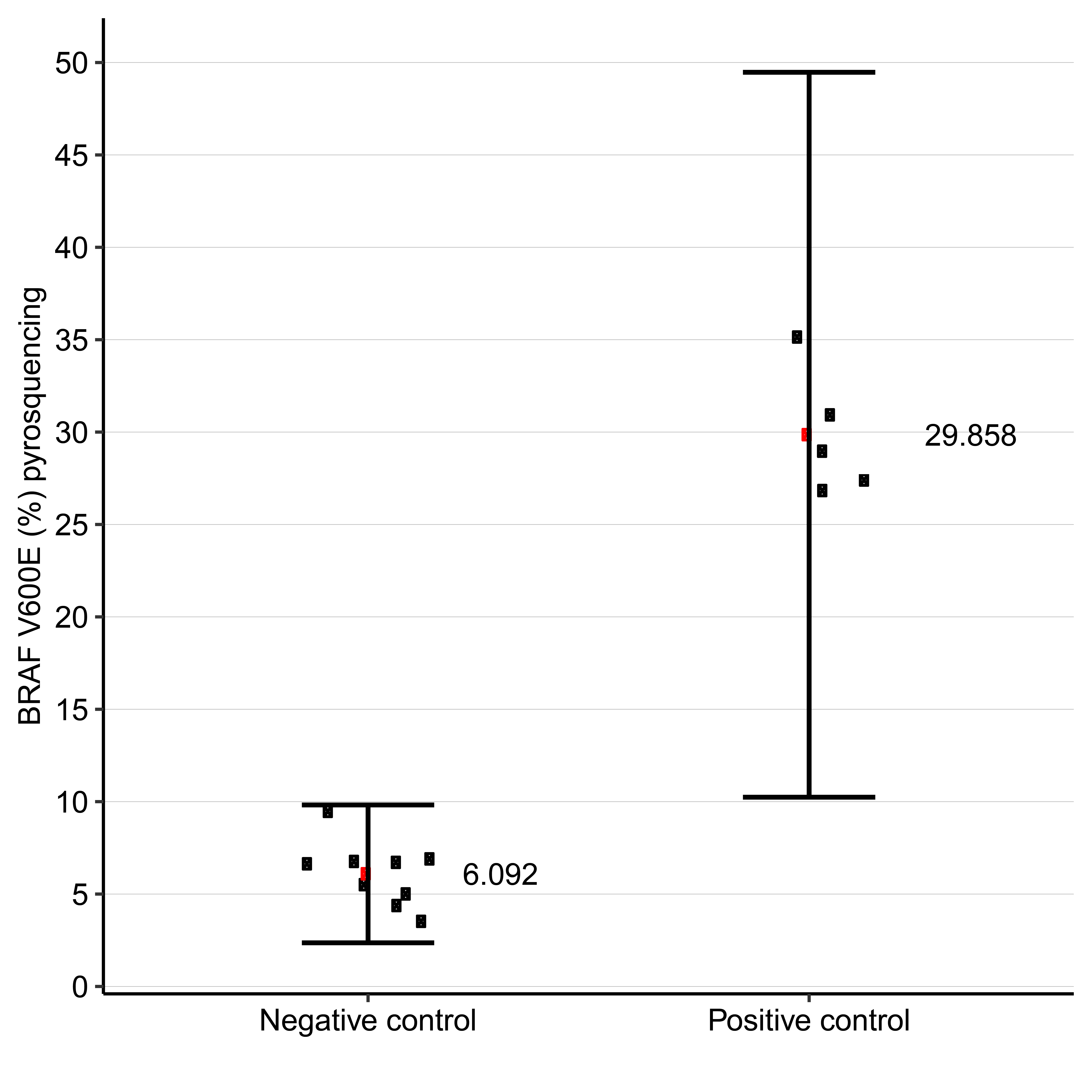
**

**Figure. S1. Percentage of all negative and positive controls obtained through pyrosequencing.** Pyrosequencing percentage of BRAF V600E is present even in the negative control. To account for this normal variability in detection SNVs in DNA derived from FFPE tissue the 99.98% confidence interval was calculated. With this it was defined the upper limit of 9.82%. Anything above this value should be considered positive, and anything under it cannot be distinguished from negative. In this method, both positive and negative controls were samples with mutational status confirmed by Sanger Sequencing.


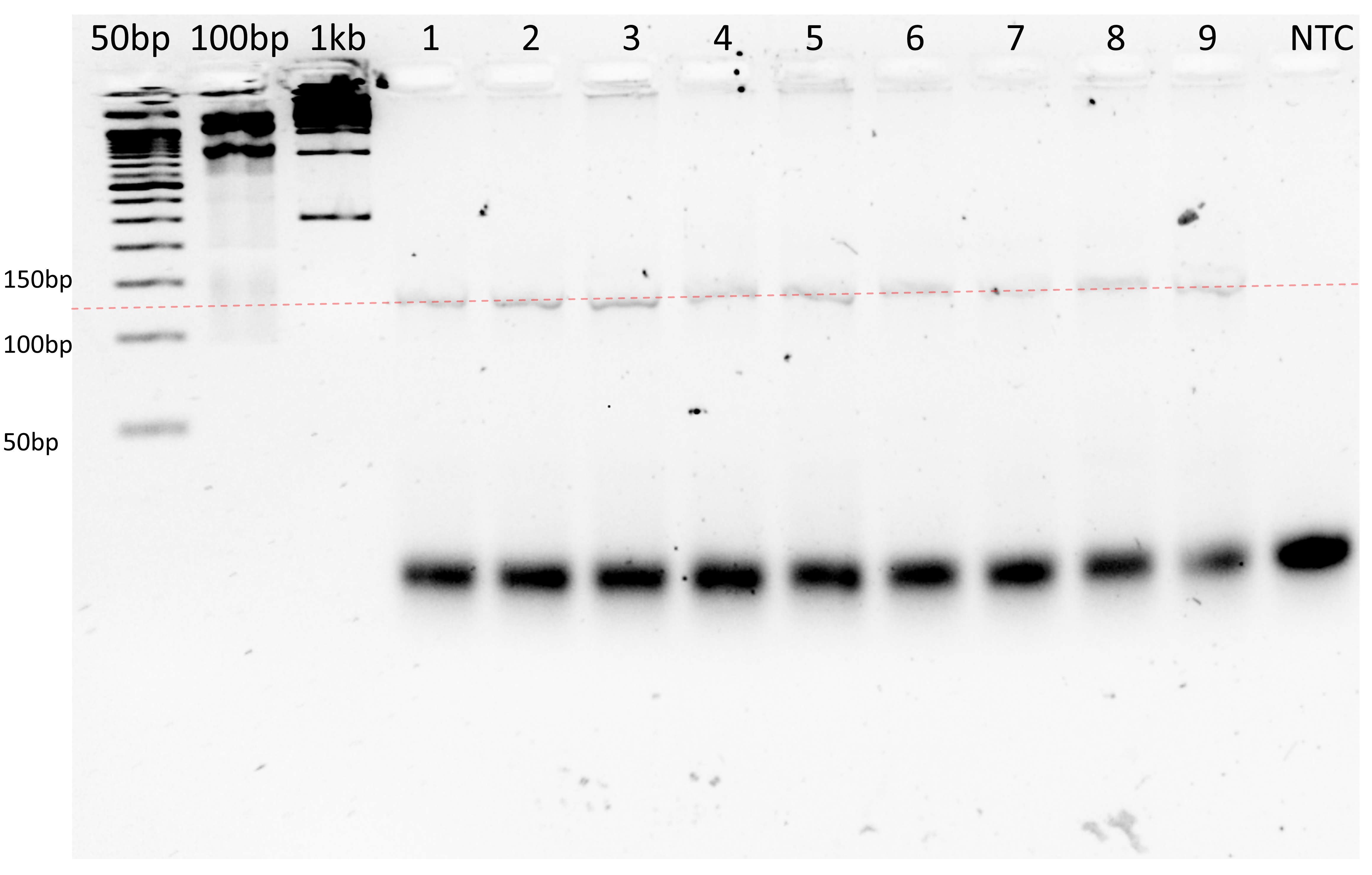


**Figure. S2. High resolution 3% agarose gel.** Wells 1 to 9 are positive controls consisting of DNA obtained from cells. By observing the 50bp molecular size ladder, it is possible to infer the size of PCR products (approximately 130bp). The gel image was obtained with Gel Doc™ EZ System and the software ImageLab with high-intensity bands exposure. The bands sizes, wells sample names and red dashed line were added post-acquisition with Adobe Photoshop to better represent the gel and band sizes. NTC = No template control, or negative control.





**Figure. S3. Digital PCR Training dataset.** This figure shows the manual classification of droplets, excluding rain droplets, to use as the training dataset for k-NN algorithm. Simple grid classification was used, which is not perfect as observed by some aberrations as seem in well B03, B04 and B05. This expected no clusters where drawn and it was all classified by using hard thresholds of channel one and channel two amplitude.

NN = No DNA; NP = positive for wild type BRAF; PN = positive for BRAF V600E; PP = positive for both wild type and mutated BRAF; Rain = droplets hard to classify, excluded from the analysis.
